# Supplementary material for: Within-host pathogen population diversity predicts treatment response in Tuberculosis
Source: medRxiv. 2026 Jun 19:2026.06.16.26355818. Preprint. [Version 1] doi: 10.64898/2026.06.16.26355818 (PMC13308281; doi:10.64898/2026.06.16.26355818)
Supplement: 1 [file NIHPP2026.06.16.26355818V1-supplement-1.pdf]

medRxiv preprint doi: https://doi.org/10.64898/2026.06.16.26355818; this version posted June 19, 2026. The copyright holder for this preprint (which was not certified by peer review) is the author/funder, who has granted medRxiv a license to display the preprint in perpetuity.
It is made available under a CC-BY 4.0 International license .

Supplement

Results

A. Identification of unfixed substitution variants from hybrid assemblies

To build a ground truth dataset of unfixed single nucleotide variants (SNVs), which we define as having a within-sample allele fraction (AF) ≥ 0.05 and AF ≤ 0.95, we performed variant calling using hybrid PacBio / Illumina assemblies as reference genomes. We selected a minimum allele fraction of 5% because variant allele fractions below 5% are more likely to be noise.

A total of 186 PacBio HiFi sequencing samples were available from this cohort. Due to research constraints, not every participant had a bacterial sample sequenced with PacBio reads. These were sequenced from sputum cultures during the first 12 weeks of TB treatment or at 5 months after starting treatment. The reads were assembled according to a previously published pipeline (**Methods**).[65] Of the 186 long read sequencing samples, 154 were assembled into a single

medRxiv preprint doi: https://doi.org/10.64898/2026.06.16.26355818; this version posted June 19, 2026. The copyright holder for this preprint (which was not certified by peer review) is the author/funder, who has granted medRxiv a license to display the preprint in perpetuity.
It is made available under a CC-BY 4.0 International license .

circular contig (**Supplementary Data 2**). These ranged in size from 4.36 to 4.44 megabase pairs (Mbp) with coverages of 14-295x (median = 89.5x) (**Supplementary Fig. 1a**). The lineage (L) distribution is 90 L2, 57 L4, six L3, and one L1.

A total of 767 culture samples from this cohort were sequenced with Illumina sequencing (**Supplementary Data 1**). For unfixed variant detection, we restricted to the 738 out of 767 Illumina samples with an F2 score ≤ 0.03 to focus on changes in monoclonal samples. 172 of the 738 samples were able to be matched to a hybrid assembly from the same participant, though not necessarily from the same culture sample. We verified that the lineage assignments[66] for the Illumina sample and the hybrid assembly matched.

Illumina reads were aligned to the personal reference genomes, followed by variant calling with freebayes. We chose freebayes because benchmarking multiple variant callers, including those optimized for low-frequency variants, on unfixed variant calling in *Mtb* showed that freebayes achieved the highest precision and recall.[26] Variants with 0.05 ≤ AF ≤ 0.95 were transferred to H37Rv coordinates using minimap2. We inspected the transfer rate of the unfixed variants for the 172 samples with personal reference genomes, and found that ten variants (9 SNVs, 1 indel) were not transferred between coordinate spaces: five are in the IS6110 transposable element[67] between H37Rv coordinates 3,710,433 and 3,713,461, and one occurs downstream of this element in the gene *sugI* (coordinates 3,717,090–3,718,598 in H37Rv). However, the start of *sugI* is predicted to be uncertain.[68] The region around *sugI* is not difficult to map reads to, and we note that this variant was picked up when Illumina reads were aligned to H37Rv. The only step that fails is the transformation of coordinates from the personal genome to H37Rv, which may be complicated by its proximity to the IS6110 element. The other four variants occur in *PPE60* (N = 3) and *PE_PGRS55* (N = 1). Both of these genes demonstrate evidence of recombination with other genes in the *Mtb* genome,[69] which makes unambiguous coordinate transfer difficult.

To obtain a final list of unfixed variants per sample, we required the coverage at each unfixed variant site to be at least half the global median coverage because short reads from especially repetitive regions can be mismapped, even when using a sample's own genome as the reference. Finally, all positions within ribosomal RNAs (*rrs, rrl,* and *rrf*), transposable elements, and phage sequences were removed. The ribosomal RNAs are prone to miscalled unfixed variants due to even small amounts of contamination with environmental bacteria because of their high degree of homology across bacteria.[70,71] A total of 401 unfixed SNVs remained. The number of unfixed SNVs per sample ranged from 0-14, with a median of 3 (**Supplementary Fig. 1b**).

B. Unfixed variant calling against the H37Rv reference genome

We used the same alignment and variant calling parameters as for the personal genomes. We filtered for candidate high quality unfixed SNVs using the following criteria: within-sample allele fraction (AF) ≥ 0.05 and AF ≤ 0.95, read depth ≥ 5, mapping quality ≥ 40, at least 2 forward and 2 reverse reads (determined by freebayes) supporting the SNV, and an average Phred quality of

medRxiv preprint doi: https://doi.org/10.64898/2026.06.16.26355818; this version posted June 19, 2026. The copyright holder for this preprint (which was not certified by peer review) is the author/funder, who has granted medRxiv a license to display the preprint in perpetuity. It is made available under a CC-BY 4.0 International license .

bases supporting the variant ≥ 20. We further excluded positions in low mappability regions or with an empirical base pair recall < 0.95,[61] and all positions in transposable elements, phage sequences, and ribosomal RNAs. The number of unfixed variants per sample begins to increase when F2 (a lineage mixing metric)[72] > 0.03 (**Supplementary Fig. 1c**), so this is how the threshold was chosen for monoclonal samples. Of the 767 Illumina samples, 738 have an F2 ≤ 0.03. The lineage distribution of the 738 samples is 366 L2, 323 L4, 48 L3, and one L1.

After the above filtering criteria, the number of unfixed SNVs per sample among the 172 samples that also have personal reference genomes ranged from 0-17, with a median of 4 (**Supplementary Fig. 1d**). This is significantly larger than the number of unfixed SNVs per sample determined using variant calling against the personal reference genomes (one-sided paired t-test $p = 4.98 \times 10^{-34}$). This indicated that despite the filtering criteria above, many false unfixed SNVs were called when aligning short reads to H37Rv. The range of the difference in the number of unfixed SNVs between variant calling with H37Rv and the personal genomes is 0-14, with an average of 2.7 false unfixed SNVs per sample.

If there is systematic bias in calling unfixed SNVs, we would expect to observe considerable differences in the genes or intergenic regions with the most unfixed SNVs. The genes or intergenic regions with the most common unfixed SNVs are *Rv2082, Rv2319c*, *iniB*, and *uspC*, with 394, 368, 237, and 234 SNVs each, respectively. *Rv2319c* appears to have a structural variant, as evidenced by the high variance in coverage and the large number of discordantly paired reads (green) (**Supplementary Fig. 2a**). We also observed false variants at regions with a large number of reads with soft clipping (**Supplementary Fig. 2b**). In this sample, the variant is fixed, but the reads that do not support the variant are soft clipped, suggesting that they originate from elsewhere in the genome. False unfixed SNVs also occur near fixed indels. Reads that do not sufficiently cover an indel do not unambiguously provide evidence for the indel, and instead, they appear to support one or more substitutions (**Supplementary Fig. 2c**).

C. Duplication of *Rv2081c-Rv2082* causes numerous false unfixed SNVs

Among samples with both long and short read sequencing, when the Illumina reads were aligned to H37Rv, we observe 56 unfixed SNVs in the gene *Rv2082* between coordinates 2,338,773 and 2,340,477 (**Supplementary Fig. 2d**) across 8 samples (6 L3 and 2 L4 samples, but the two L4 samples come from a single individual). None of these was found when aligning Illumina reads to the personal genomes for these samples. Suspecting a structural variant here, we aligned PacBio reads to H37Rv and performed structural variant detections with sniffles. Sniffles identifies 2.6 or 5.3 kilobase pair (kbp) insertions in this region, as evidenced by the large increase in coverage between positions 2,338,129 and 2,340,759 in H37Rv, which covers most of *Rv2081c* and *Rv2082* (**Supplementary Fig. 3c**).

We extracted the regions between the start of *Rv2081c* and the beginning of *Rv2083* for the 8 samples. The lengths of these regions are 5,466 base pairs for the L3 samples and 8,111 base pairs for the L4 samples. We then performed individual pairwise alignments between the 8 sample sequences to H37Rv. Based on the aforementioned lengths, we suspected that there

medRxiv preprint doi: https://doi.org/10.64898/2026.06.16.26355818; this version posted June 19, 2026. The copyright holder for this preprint (which was not certified by peer review) is the author/funder, who has granted medRxiv a license to display the preprint in perpetuity.
It is made available under a CC-BY 4.0 International license .

are two extra copies of this region in L4 and one extra copy in L3. Therefore, we concatenated the H37Rv sequence to itself twice before aligning to the L3 samples and concatenated the H37Rv sequence to itself thrice to align it to the L4 samples. For the L3 samples, the alignments are all 5,633 nucleotides with 5,097 (90.5%) exact matches. For the L4 samples, the alignments are of length 8,380 nucleotides with 7,626 (91.0%) exact matches. This suggests that there is one additional paralog in L3 and two additional paralogs in L4. The L4 samples belong to L4.1.2, and they are the only samples of this L4 sublineage with PacBio sequencing that have this duplication. The large number of SNVs within the duplicated regions indicate sequence divergence between the paralogs of this region or mismapping due to the repetitive nature of this region (**Supplementary Fig. 3a-b**).

To investigate this duplication in the rest of the short-read sequencing sample set, we called structural variants from short read sequencing alignments to H37Rv using delly. We identified structural variants with length ≥ 2,500 base pairs and at least 10 paired end reads of support in 34 participants' baseline samples, all of which are in lineages 3 and 4. The sublineage distribution of lineage 4 samples is four L4.1.2, one L4.2.2, and one L4.1 (**Supplementary Fig. 3d**). L4.1.2 and L4.2.2 are monophyletic, while the L4.1 sample is farther away. The clustering of this duplication in phylogenetic space suggests that it arose multiple times in different subclades and then was propagated through transmission. A larger long read sequencing sample set with a wider lineage distribution (in particular, L1 and L7) is needed to develop hypotheses for the phylogenetic history of this duplication.

D. Logistic regression model to predict the accuracy of an unfixed SNV

Given the observations about reference bias-induced false positive unfixed variant calls, we built a logistic regression model to predict the probability of a candidate unfixed SNV being accurate using the following features:

- **Discordant reads:** Number of discordantly paired reads normalized to coverage
- **Base quality:** Average base quality of bases supporting the variant. Most reads at the site of an unfixed variant support the reference allele, so the average base quality computed by variant callers reflects the qualities of both reference and alternate alleles.
- **Coverage:** Ratio of coverage at the site to the rolling average
- **Strand bias:** Absolute value of the difference between 0.5 and the proportion of reads supporting the variant that are in the forward orientation
- **Soft clipped bases:** Number of soft clipped bases at the variant site normalized to coverage
- **Soft clipped reads:** Ratio of reads supporting a variant that are soft clipped to the total number of reads supporting the variant. This is different from the above variable because reads can be soft clipped elsewhere, not just at the site with the variant.
- **Intra-read variant position:** Median of the normalized position within reads that support the variant. The closer the variant is to the edge of the read, the smaller this value is.

medRxiv preprint doi: https://doi.org/10.64898/2026.06.16.26355818; this version posted June 19, 2026. The copyright holder for this preprint (which was not certified by peer review) is the author/funder, who has granted medRxiv a license to display the preprint in perpetuity.
It is made available under a CC-BY 4.0 International license .

The coverage rolling average was computed with a window size of 100 base pairs, and we took the maximum of the rolling averages computed from the up- and downstream directions. Because we expected that increases and decreases in coverage have different effect sizes on the probability of a variant being real (*i.e.,* a drop in coverage is likely more indicative of a false unfixed SNV than an increase in coverage), we split the coverage ratio variable into two variables, one for ratios ≥ 1 and one for ratios < 1.

After first excluding all variants in *Rv2081c* and *Rv2082* and the intergenic region, which spans positions in the range [2338065, 2340874] in H37Rv, the model was trained on 802 candidate unfixed SNVs (401 real, 401 not real) from the 172 WGS samples with matched personal reference genomes. The variants and associated statistics were derived from variant calling against H37Rv, and the real labels were derived from their presence or absence when variant calling with the personal reference genomes.

Because the model is focused on accuracy for this dataset, rather than generalizability, we trained a single model on all 802 variants. Predictions were then obtained for 2,366 additional unfixed SNVs from 565 WGS samples without personal reference genomes. Five of the eight estimated odds ratios were significant at a significance level of $\alpha = 0.05$, and the effect sizes were consistent with expectation, with odds ratios > 1 for base quality and intra-read variant position, and odds ratios < 1 for the others (**Supplementary Table 3**). Of the 2,366 candidate unfixed SNVs in the 565 WGS samples without personal reference genomes, 1,205 were predicted to be real (**Supplementary Data 4**). On the train set, the classification statistics are as follows: area under the curve (AUC) = 0.991, precision = 0.978, and recall = 0.978. There are 9 false negatives and 9 false positives.

The distributions of the number of unfixed SNVs per sample detected from the personal reference genomes and detected from H37Rv are not significantly different by a two-sided Kolmogorov-Smirnov test (p = 0.96), while the distributions were significantly different ($p = 3.9 \times 10^{-23}$) before filtering the H37Rv-called unfixed SNVs using the logistic regression model. 224/738 (30%) samples of the full dataset and 45/172 (26%) samples of the ground truth dataset have no unfixed SNVs, while only 38/738 (5.1%) of samples had 0 unfixed SNVs before filtering.

E. Adjusting indel allele fractions to exclude false positives

Calling unfixed indels is generally less affected by reference bias because variant callers require greater evidence to call indels than SNVs. The most salient issue in accurately calling low frequency indels is reads not sufficiently covering an indel, especially in repetitive regions, which are common in the *Mtb* genome. These reads artificially bring down the allele fraction of the indel, making it appear as though a fixed indel is unfixed. We excluded reads found too close to the candidate indel and reads with soft clipping because these are unable to support an indel, even if it is fixed (**Methods**). After running the pipeline, each indel has an adjusted allele fraction computed using only reads that sufficiently span the indel site.

medRxiv preprint doi: https://doi.org/10.64898/2026.06.16.26355818; this version posted June 19, 2026. The copyright holder for this preprint (which was not certified by peer review) is the author/funder, who has granted medRxiv a license to display the preprint in perpetuity.
It is made available under a CC-BY 4.0 International license .

We performed similar benchmarking using the 172 available hybrid assemblies. We compared unfixed indels, those with 0.05 ≤ AF ≤ 0.95 called using H37Rv as the reference genome against those called using the personal assemblies as individual references. A single unfixed indel was not able to be transferred from a personal genome to H37Rv coordinates, but it occurs 4 base pairs upstream of the IS6110 element between *Rv3324c* and *Rv3328c*, where pairwise genome alignment is ambiguous. A total of 67 true indels were called across the 172 samples with matched personal genomes. The number of indels per sample ranges from 0-4 with an average of 0.39. A total of 2,724 unfixed indels were called from these samples after variant calling against H37Rv (67 true positives and 2,657 false positives). After adjusting the AFs and excluding indels with an AF > 0.95, only 3 false positives remained.

After aligning short reads to H37Rv and performing the same quality control as in **Methods Section B,** 6,342 unfixed indels were identified in the full set of 738 WGS samples. The number of unfixed indels ranged from 0-18 with an average of 8.6 per sample, which is significantly larger than the distribution determined from the personal reference genomes (p = 0, Welch's t-test). After running the above pipeline on the full dataset of 738 samples, there are only 278 total unfixed indels (**Supplementary Data 7**). The number of unfixed indels ranges from 0-5, with an average of 0.38 unfixed indels per sample, which is not significantly different from the distribution of unfixed indels determined on the personal reference genomes (p = 0.42, two-sided Welch's t-test). There is no significant difference in the number of unfixed indels per sample across the four lineages (p = 0.32, likelihood ratio test). Two-thirds of the unfixed indels are frameshifts (N = 186). The remainder consists of 40 inframe deletions, 17 inframe insertions, and 35 intergenic variants.

F. Additional unfixed variant burden associations

In Kaplan Meier analysis, we observed a non-monotonic relationship between baseline pathogen diversity and the risk of outcomes (**Fig. 4b**). A boundary knot of 3 was found to minimize the Akaike Information Criterion (AIC) (**Supplementary Fig. 5a**). We therefore fit different hazard ratios below and above an unfixed variant count of 3 in Cox proportional hazards models. Adding unfixed variant burden at baseline to the multivariate model increases the concordance index from 0.68 to 0.73, reduces the AIC from 278.1 to 276.0, and significantly increases the log-likelihood (likelihood ratio test p-value = 0.01). The Cox-Snell pseudo R^2, indicates that unfixed variant burden explains an additional 1.7% in the variation in outcome over the base model with patient covariates only.

G. Unfixed variant burden at baseline is a better predictor of composite outcomes than unfixed variant burden at follow-up

To compare model fits between unfixed variant burdens at baseline and follow-up, we performed the analysis on the 262/364 individuals with longitudinal sequencing. We used boundary knots of 1 for baseline and 2 for follow-up to maximize the AIC in each case (**Supplementary Fig. 5c-d**). Unfixed variant burden at baseline increases the concordance index from 0.72 to 0.77, lowers the AIC from 216.5 to 212.5, and explains an additional 3.0% of the variation in outcome

medRxiv preprint doi: https://doi.org/10.64898/2026.06.16.26355818; this version posted June 19, 2026. The copyright holder for this preprint (which was not certified by peer review) is the author/funder, who has granted medRxiv a license to display the preprint in perpetuity.
It is made available under a CC-BY 4.0 International license .

time, whereas unfixed variant burden at follow-up has concordance index = 0.74 and AIC = 216.2 and explains 1.6% of additional variation. These results demonstrate that the relationship between unfixed variant burden and composite outcomes is robust to the sampling time when samples are taken within the first 12 weeks since treatment initiation.

H. Unfixed variant burden does not associate with time to culture conversion

We additionally tested if unfixed variant burden associates with time to negative culture conversion (TCC). Sputum was sampled weekly for the first 12 weeks of treatment. TCC was defined as the number of weeks between the time of treatment initiation and the first of two consecutive *Mtb*-negative sputum cultures not followed by another positive culture. To be included in the TCC analysis, participants had to have at least 3 uncontaminated sputum samples and a positive *Mtb* culture within the first 5 weeks of initial screening, leaving 323/364 patients remaining for the TCC analysis. The median TCC is 9 weeks.

The TCC Cox proportional hazards model was stratified by 4 patient covariates – previous TB disease, underweight (*i.e.,* BMI < 18), smear positivity, and PLI > 25% (**Supplementary Fig. 6a-d**) – in order to satisfy the Cox proportional hazards assumption. The directions of these effects generally agree with expectation, and the log-rank test indicates significantly different Kaplan-Meier (KM) estimates, with previous TB (p = 0.11), underweight (p = 0.017), smear positivity ($p = 3.3 \times 10^{-8}$), and PLI > 25% ($p = 6.6 \times 10^{-5}$) being associated with longer TCC. After these stratifications, only age and TTP at baseline are associated with longer TCC. Unfixed variant burden is not associated with TCC (**Supplementary Fig. 6e**).

I. Mixed infections are not associated with worse outcomes or TCC

However, in this dataset, mixed infections, either represented by F2 score or binarized at 0.03, were not associated with worse outcomes over clonal infections. In univariate models, F2 score had an aHR of 1.19 (95% CI 0.64 - 2.20, p = 0.58), and the binary variable F2 > 0.03 had an aHR of 1.54 (95% CI 0.36 - 6.50, p = 0.56). The prevalence of mixed infections at baseline is low (5%), and therefore the dataset may be underpowered to detect an association if it exists.

Tables

ID	Baseline		Follow-Up		Group
	Major Lineage (%)	Minor Lineages (%)	Major Lineage (%)	Minor Lineages (%)	
T0010	4.1.1.1 (54)	2.2.M1.1 (46)	2.2.M1.1 (100)		Major lost

medRxiv preprint doi: https://doi.org/10.64898/2026.06.16.26355818; this version posted June 19, 2026. The copyright holder for this preprint (which was not certified by peer review) is the author/funder, who has granted medRxiv a license to display the preprint in perpetuity.
It is made available under a CC-BY 4.0 International license .

T0066	2.2.M1.1 (90)	4.3.2.1 (10)	2.2.M1.1 (100)		Minor lost
T0072	4.1.2.1 (59)	4.8 (41)	4.1.21 (100)		Minor lost
T0087	4.1.2.1 (57)	4.8 (43)	4.8 (100)		Major lost
T0116	2.2.M1.1 (95)	4.3.3 (5)	2.2.M1.1 (100)		Minor lost
T0152	4.3.3 (85)	4.3.4.2.1 (15)	4.3.4.2.1 (100)		Major lost
T0167	2.2.M2.2 (82)	4.1.1.3 (18)	2.2.M2.2 (74)	4.1.1.3 (26)	Persistent mixed
T0198	2.2.M1.1 (89)	4.4.1.1 (11)	2.2.M1.1 (100)		Minor lost
T0204	2.2.M4.4 (86)	3.1.1.1 (14)	2.2.M4.4 (70)	3.1.1.1 (30)	Persistent mixed
T0224	2.2.M4.4 (99)	2.2.M1.1 (1)	2.2.M1.1 (58)	2.2.M4.4 (42)	Persistent mixed
T0235	2.2.M1.1 (100)		4.8 (88)	2.2.M1.1 (12)	Additional lineage
T0253	4.8 (98)	2.2.M1.1 (2)	2.2.M1.1 (100)		Major lost
T0267	2.2.M1.1 (76)	4.3.2.1 (24)	4.3.2.1 (100)		Major lost
T0305	4.3.2.1 (58)	2.2.M1.1 (42)	4.3.2.1 (100)		Minor lost
T0311	2.2.M1.1 (63)	4.3.2.1 (36), 4.3.3 (1)	2.2.M1.1 (100)		Minor lost
T0322	4.1.1.3 (93)	2.2.M1.1 (7)	4.1.1.3 (100)		Minor lost
T0364	2.2.M4.4 (80)	2.2.M1.1 (20)	2.2.M4.4 (100)		Minor lost
T0367	2.2.M1.1 (87)	3.5.1 (13)	3.5.1 (100)		Major lost
T0393	2.2.M1.1 (93)	4.1.1.3 (7)	2.2.M1.1 (97)	4.1.1.3 (3)	Persistent mixed
T0427	2.2.M4.8 (78)	2.2.M1.1 (15), 4.3.4.2.1 (7)	2.2.M1.1 (100)		Major lost
T0440	2.2.M4.8 (97)	2.2.M4.4 (3)	2.2.M4.4 (100)		Major lost

medRxiv preprint doi: https://doi.org/10.64898/2026.06.16.26355818; this version posted June 19, 2026. The copyright holder for this preprint (which was not certified by peer review) is the author/funder, who has granted medRxiv a license to display the preprint in perpetuity.
It is made available under a CC-BY 4.0 International license .

T0441	2.2.M1.1 (85)	4.1.2.1 (15)	2.2.M1.1 (97)	4.1.2.1 (3)	Persistent mixed
T0444	4.3.4.2.1 (69)	4.1.1.1 (31)	4.1.1.1 (100)		Major lost
T0457	2.2.M1.1 (96)	4.3.4.2.1 (4)	2.2.M1.1 (100)		Minor lost
T0465	3.1.3.1 (98)	2.2.M1.1 (2)	3.1.3.1 (100)		Minor lost

Supplementary Table 1. Lineage proportions of culture samples of 25 participants with mixed infections at either baseline or follow-up. Mixed infections are those with multiple lineages detected with a prevalence of at least 1% by the TBtypeR tool.[62] Both timepoints occur during the first 12 weeks of enrollment. Only one of these participants had a sequenced culture at month 5: pid T0204 had a 100% L2.2.M4.4 sample then. The participants are grouped into categories describing changes relative to baseline. All lineage 2 samples in this cohort are part of the L2.2.1 sublineage, which is further divided into subclades.[64]

ID	Variant	Baseline		Follow-Up		Interval (weeks)
		AF	Lineages	AF	Lineages	
T0116	inhA_c.-154G>A	92%	2.2.M1.1 (95%), 4.3.3 (5%)	100%	2.2.M1.1	5
T0305	inhA_p.Ser94Ala	56%	4.3.2.1 (58%), 2.2.M1.1 (42%)	100%	4.3.2.1	7
T0322	katG_p.Ser315Thr	96%	4.1.1.3 (93%), 2.2.M1.1 (7%)	100%	4.1.1.3	4
	rpsL_p.Lys43Arg	93%		100%		
T0441	inhA_c.-777C>T	84%	2.2.M1.1 (85%), 4.1.2.1 (15%)	96%	2.2.M1.1 (97%), 4.1.2.1 (3%)	7
	rpsL_p.Lys88Arg	86%		97%		

Supplementary Table 2. Within-sample allele frequencies of known resistance mutations in participants with mixed infections. The two variants in *rpsL* are associated with streptomycin resistance, and the other four variants are associated with isoniazid resistance.[2] All four individuals had phenotypically measured isoniazid resistance at baseline. Phenotypic streptomycin resistance was not measured in this cohort.

Variable	p-value	Odds Ratio (OR)	95% confidence interval of OR
Intra-read position (closer to middle)	4.24×10^{-14}	11.1	5.93 – 20.6

medRxiv preprint doi: https://doi.org/10.64898/2026.06.16.26355818; this version posted June 19, 2026. The copyright holder for this preprint (which was not certified by peer review) is the author/funder, who has granted medRxiv a license to display the preprint in perpetuity.
It is made available under a CC-BY 4.0 International license .

Discordant reads	3.58×10^{-10}	0.003	4.91×10^{-4} – 0.018
Soft clipped bases	2.31×10^{-9}	6.37×10^{-48}	2.09×10^{-63} – 1.94×10^{-32}
Strand bias	2.29×10^{-5}	0.325	0.193 – 0.546
Soft clipped reads	0.013	0.415	0.208 – 0.828
Average base quality	0.094	1.60	0.924 – 2.75
Coverage increase	0.110	0.744	0.518 – 1.07
Coverage decrease	0.274	0.452	0.109 – 1.87

Supplementary Table 3. Odds ratios and p-values in the logistic regression model. Variables are ordered by increasing p-value. Confidence intervals are Wald intervals.

**Mutation**	**Type**	**Rate in BL Samples**	**Rate in FU Samples**
C > T	Transition	1.17×10^{-4}	1.04×10^{-4}
A > G	Transition	1.15×10^{-4}	9.49×10^{-5}
G > A	Transition	1.11×10^{-4}	9.07×10^{-5}
T > C	Transition	1.07×10^{-4}	1.07×10^{-4}
A > C	Transversion	5.93×10^{-5}	4.09×10^{-5}
C > A	Transversion	4.41×10^{-5}	2.97×10^{-5}
T > G	Transversion	4.22×10^{-5}	3.30×10^{-5}
G > T	Transversion	3.53×10^{-5}	3.05×10^{-5}
C > G	Transversion	3.10×10^{-5}	2.28×10^{-5}
G > C	Transversion	2.28×10^{-5}	1.94×10^{-5}
A > T	Transversion	1.19×10^{-5}	6.59×10^{-6}
T > A	Transversion	6.59×10^{-6}	3.96×10^{-6}

Supplementary Table 4. Empirically estimated unfixed mutation rates for each substitution at baseline (BL) and follow-up (FU). The mutations are ordered by decreasing rate in baseline samples. The ratio of average transition rate to average transversion rate is 3.54 at baseline and 4.20 at follow-up. These values were used to determine the expected rate of each type of substitution to parameterize the Poisson test.

medRxiv preprint doi: https://doi.org/10.64898/2026.06.16.26355818; this version posted June 19, 2026. The copyright holder for this preprint (which was not certified by peer review) is the author/funder, who has granted medRxiv a license to display the preprint in perpetuity.
It is made available under a CC-BY 4.0 International license .

Region	Function	Indel	AF Change	Lineages
Upstream of *Rv0759c*	Unknown	854,252 delC<br>854,252 insC	Many	2, 4
Rv1190	Unknown	1,333,661 insG<br>1,333,661 insG<br>1,333,661 insG<br>1,333,661 insGG<br>1,333,661 insGG<br>1,333,661 insGG	0 → 28%<br>0 → 8%<br>0 → 15%<br>95 → 0%<br>88 → 0%<br>90 → 97%	2
Upstream of *epsR*	ESX-1 transcriptional regulator of *espACD*	4,323,354 insG	71 → 99%<br>32 → 99%	2
PE_PGRS14	Unknown	928,285 delC	0 → 7.7%	3
glpK	Glycerol kinase	4,139,183 insC	6.3 → 0%	4
Rv0954	Cell division	1,066,020 delG	1.3 → 6.4%	4
espK	ESX-1 protein	4,358,979 insG	5.6 → 0%	3
Upstream of *Rv1990A*	Dehydrogenase, possible pseudogene	2,234,247 insG	7.3 → 0%	2
aspB	Aminotransferase	4,007,271 delG	73 → 0%	4
frdB	Reductase	1,760,164 insGGG	86% at baseline	2
Rv1894c	Unknown	2,141,408 insG	0 → 9%	2

Supplementary Table 5. Changes in unfixed indels in homopolymeric tracts of a single nucleotide repeated at least 7 times. The change in allele frequency of each variant is given in the "AF Change" column. Multiple values are in different individuals. The allele fractions of variants present below the lower limit of 5% were manually estimated from the alignment. The time between paired WGS samples for each participant in the table ranges from 5 to 7 weeks. With the exception of the variants in *Rv0759c, espR,* and *Rv1190*, all other variants occur in a single participant each. Because there are 12 samples (11 participants) with variants in *Rv0759c*, individual allele fractions are not listed. The individual with the *frdB* variant only had a baseline sample sequenced.

medRxiv preprint doi: https://doi.org/10.64898/2026.06.16.26355818; this version posted June 19, 2026. The copyright holder for this preprint (which was not certified by peer review) is the author/funder, who has granted medRxiv a license to display the preprint in perpetuity.
It is made available under a CC-BY 4.0 International license .

Figures

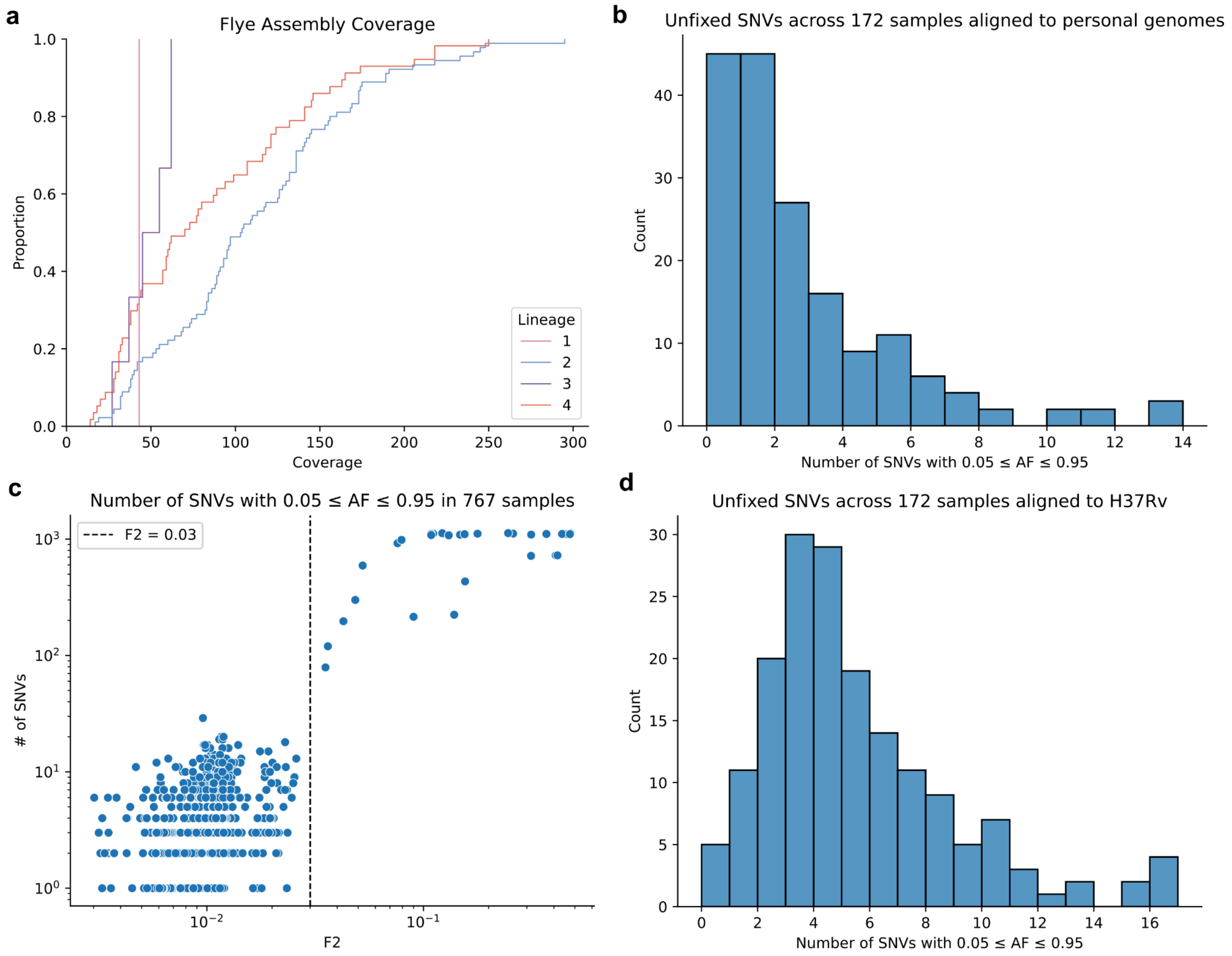

<u>Supplementary Figure 1.</u> Hybrid assembly coverage statistics and comparison of unfixed SNVs called from personal reference genomes and H37Rv. **a**: Assembly coverage based on Flye of 154 high quality hybrid assemblies. **b**: Distribution of number of unfixed single nucleotide variants (SNVs) per sample for 172 Illumina samples aligned to 154 personal reference genomes. **c**: Number of unfixed SNVs per sample vs. F2 strain mixing metric for all 767 Illumina samples aligned to H37Rv. **d**: Distribution of number of unfixed SNVs per sample for 172 samples in panel **b** but variants called against H37Rv. The total number of SNVs in panel **b** is 401, and the total number of SNVs in panel **d** is 866.

medRxiv preprint doi: https://doi.org/10.64898/2026.06.16.26355818; this version posted June 19, 2026. The copyright holder for this preprint
(which was not certified by peer review) is the author/funder, who has granted medRxiv a license to display the preprint in perpetuity.
It is made available under a CC-BY 4.0 International license .

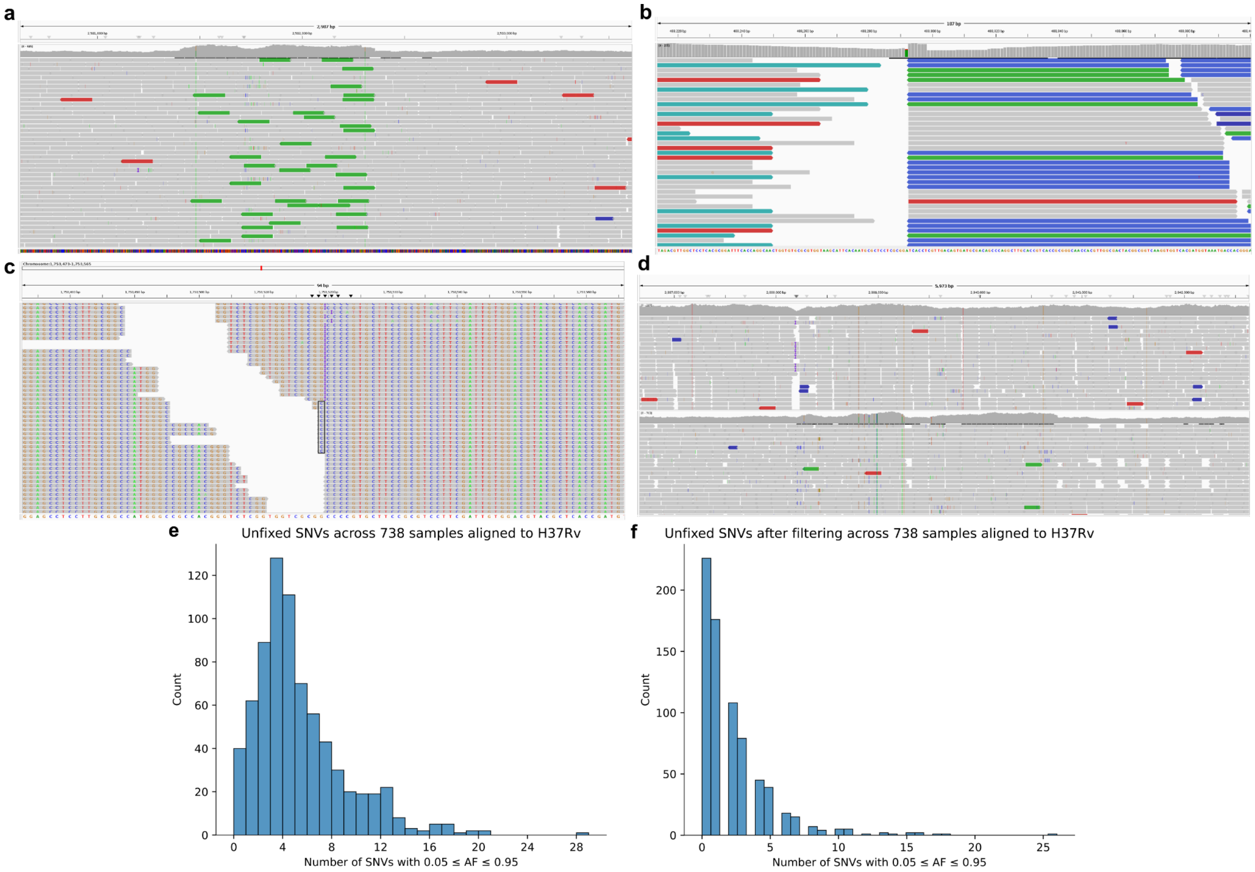

<u>Supplementary Figure 2.</u> **Examples of false positive unfixed variants used to determine relevant alignment statistics**. **a-c**: Examples of alignments of Illumina reads to H37Rv, showing reference bias-associated problems of discordantly paired reads (**a**), soft clipping (**b**), and proximity to indels (**c**). In **c**, the reads that support a G>C substitution just before the indel are boxed. **d**: Alignments of Illumina reads to H37Rv for the gene *Rv2082* for two samples: the top sample is L2.2.1, and the bottom sample is L3. The L3 sample shows a large number of unfixed SNVs in a region of increased coverage. **e**: Distribution of number of unfixed SNVs per sample for 738 samples before logistic model filtering. **f**: Distribution of number of unfixed SNVs per sample for 738 samples after all filtering and exclusion steps.

medRxiv preprint doi: https://doi.org/10.64898/2026.06.16.26355818; this version posted June 19, 2026. The copyright holder for this preprint (which was not certified by peer review) is the author/funder, who has granted medRxiv a license to display the preprint in perpetuity.
It is made available under a CC-BY 4.0 International license .

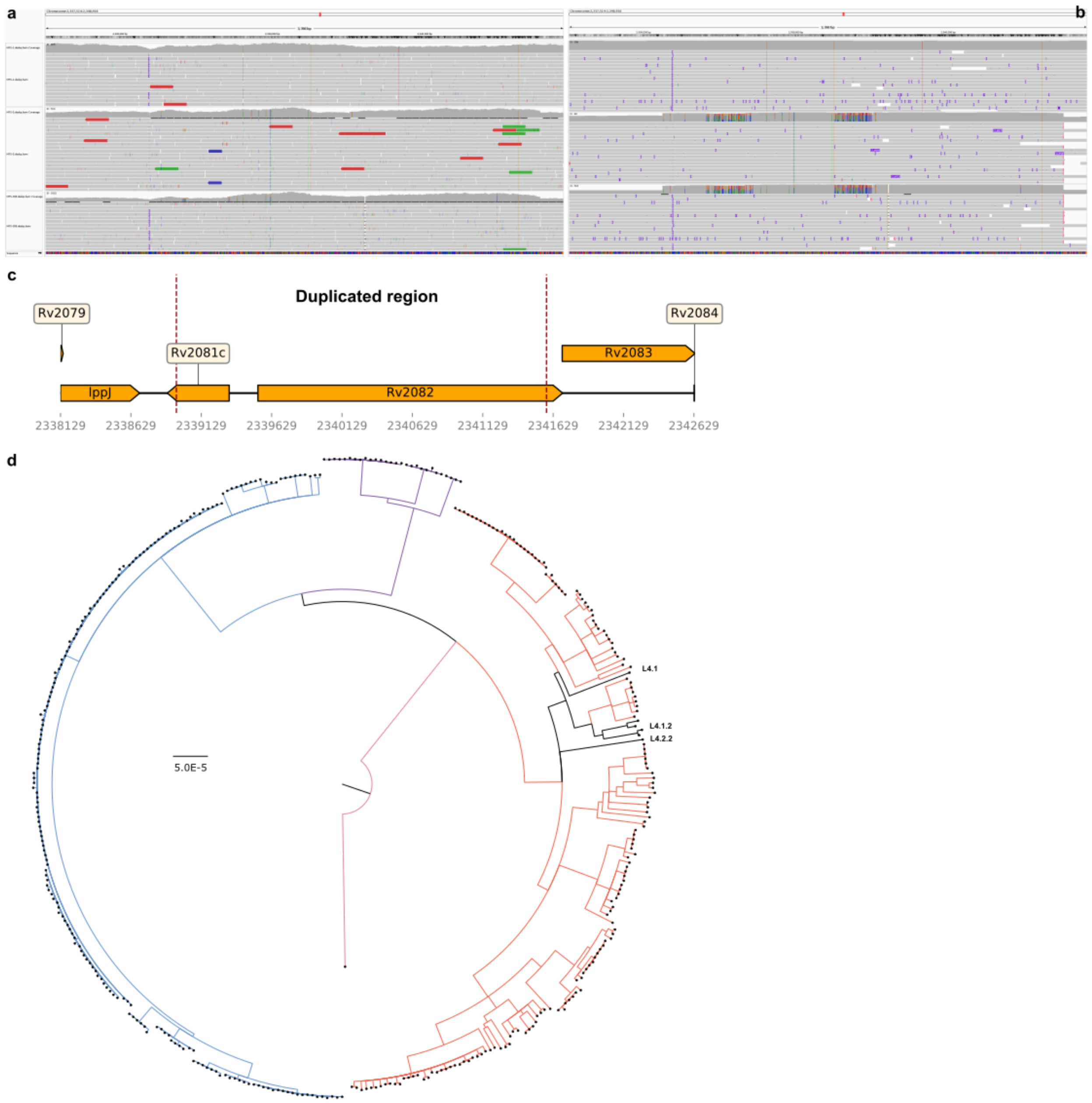

<u>Supplementary Figure 3.</u> *Rv2081c-Rv2082* duplication in lineage 4 sublineages and lineage 3. a: Short reads aligned to the H37Rv reference genome for L2 (top), L3 (middle), and L4 (bottom) samples. **b**: Long reads aligned to H37Rv for the same three samples in the same order as in **a. c:** Schematic of the duplicated region in H37Rv. The dashed red lines are the boundaries of the duplication. **d**: Maximum likelihood tree of 395 Illumina samples constructed using 14,049 genome-wide single nucleotide variants. Red = L4, purple = L3, blue = L2, pink = L1. All 28 L3 samples and 6 L4 samples have the duplication. The 6 L4 samples with the duplication are colored in black and annotated on the right side of the tree.

medRxiv preprint doi: https://doi.org/10.64898/2026.06.16.26355818; this version posted June 19, 2026. The copyright holder for this preprint (which was not certified by peer review) is the author/funder, who has granted medRxiv a license to display the preprint in perpetuity.
It is made available under a CC-BY 4.0 International license .

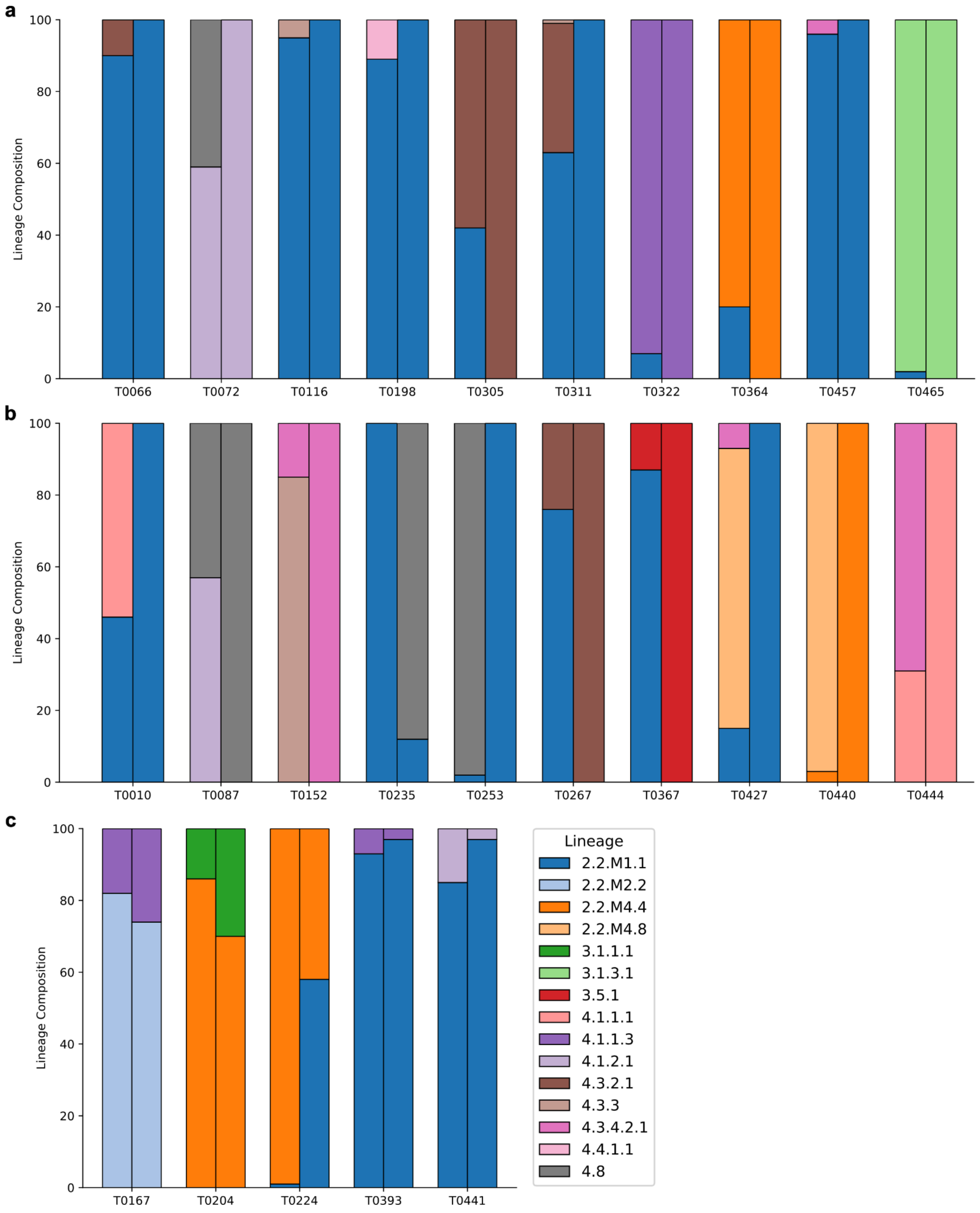

<u>Supplementary Figure 4.</u> Lineage fraction changes for 25 participants with mixed infections at baseline or follow-up. a: 10 participants in whom the major lineage at baseline remained the major lineage at follow-up. **b:** 9 participants in whom the minor lineage at baseline

medRxiv preprint doi: https://doi.org/10.64898/2026.06.16.26355818; this version posted June 19, 2026. The copyright holder for this preprint
(which was not certified by peer review) is the author/funder, who has granted medRxiv a license to display the preprint in perpetuity.
It is made available under a CC-BY 4.0 International license .

became the major lineage at follow-up and one participant (T0235), who had a single lineage at baseline, which became the minor lineage at follow-up. **c:** 5 participants who had mixed infections at both baseline and follow-up. The lineage legend is the same for all three panels.

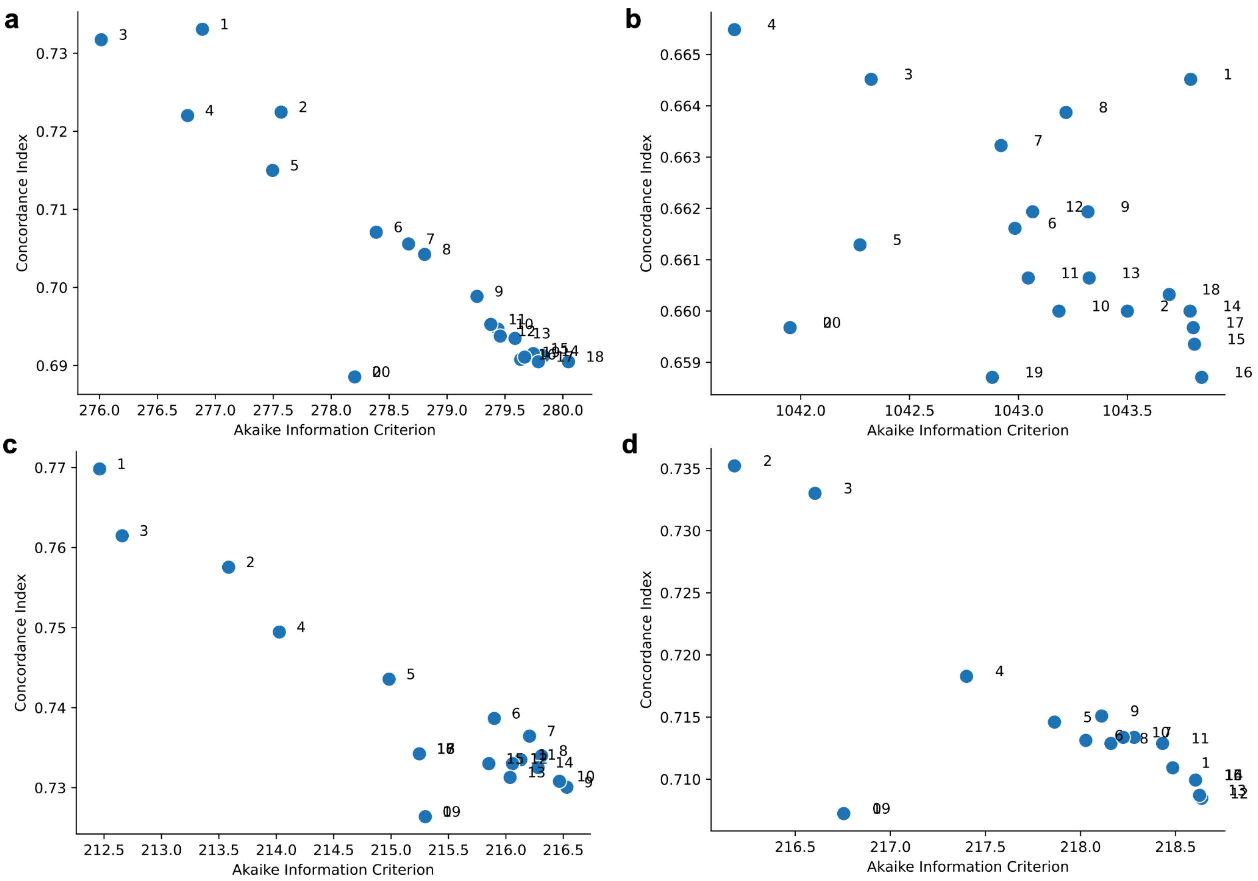

<u>Supplementary Figure 5.</u> Scatterplots of Concordance Index vs. Akaike Information Criterion to select boundary knots for unfixed variant burden splines. Different panels are different multivariate Cox proportional hazards models with unfixed variant burdens at either baseline or follow-up. **a:** Unfixed variant burden at baseline in a Cox model predicting time to unfavorable outcome for 364 participants. **b:** Unfixed variant burden at baseline in a model predicting time to culture conversion for 323 participants. **c:** Unfixed variant burden at baseline in a Cox model predicting time to unfavorable outcome for 262 participants with longitudinal sequencing. **d:** Unfixed variant burden at follow-up in a Cox model predicting time to unfavorable outcome for 262 participants with longitudinal sequencing.

medRxiv preprint doi: https://doi.org/10.64898/2026.06.16.26355818; this version posted June 19, 2026. The copyright holder for this preprint (which was not certified by peer review) is the author/funder, who has granted medRxiv a license to display the preprint in perpetuity.
It is made available under a CC-BY 4.0 International license .

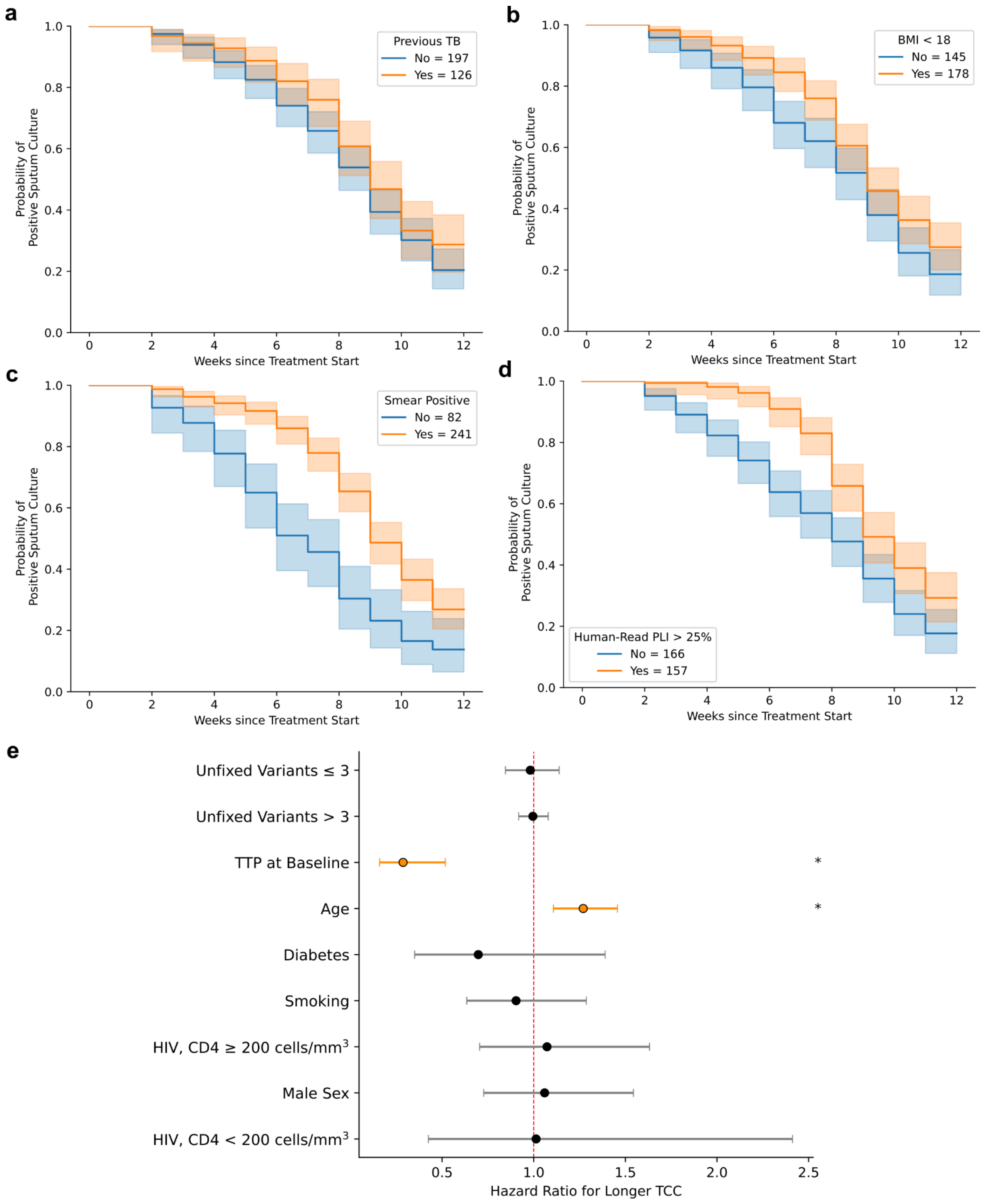

<u>Supplementary Figure 6.</u> Cox proportional hazards model for time to culture conversion (TCC) for 323 participants with clonal *Mtb* infections. **a-d**: Kaplan-Meier estimates stratified by 5 variables to satisfy the proportional hazards assumption. In order, previous TB **(a)**, underweight (BMI < 18) **(b)**, smear positivity **(c)**, PLI > 25% **(d)**. **e**: Forest plot of hazard ratios

medRxiv preprint doi: https://doi.org/10.64898/2026.06.16.26355818; this version posted June 19, 2026. The copyright holder for this preprint (which was not certified by peer review) is the author/funder, who has granted medRxiv a license to display the preprint in perpetuity.
It is made available under a CC-BY 4.0 International license .

between TCC, patient covariates, and unfixed variant burden at baseline, with 95% confidence intervals (Wald). Standard errors were computed by the Cox regression fitter in lifelines. Variables with two-sided p-values < 0.05 are shown in orange and denoted by an asterisk. All reported hazard ratios are associations with longer TCC. The hazard ratio for age is per 10 year increase, the hazard ratio for unfixed variant burden is per 10 variant increase.

Data

Supplementary Data 1: **767 Illumina sequencing samples and sequencing information**. Each sample (denoted by its MFS- ID in the "SampleID" column) has the associated participant ID ("pid"), a more descriptive sample ID ("Original_ID"), timepoint ("Sampling_Week"), and statistics about contamination, sequencing depth, lineage mixing, and lineage classification. The lineage classifications were determined by TBtypeR.[62] "mix_phylotypes" is a comma-separated list of the sublineages; "mix_props" is a comma-separated list of the proportions that each lineage in "mix_phylotypes" composes, in the same order; and "n_phy" is the total number of lineages detected at ≥ 1%. Each value in "Original_ID" is composed of the participant ID (with an "S" instead of a "T") and the sampling week. Timepoint suffixes of "m5" and "PM12" indicate month 5 since enrollment and post-treatment month 12, respectively.

Supplementary Data 2: **Hybrid assembly characteristics and short-read samples matched to hybrid assemblies.** Sheet 1: 154 complete, high-quality *Mtb* assemblies generated from PacBio HiFi sequencing and polished with matched Illumina sequencing. Lineage classifications made by fast-lineage-caller.[73] Sheet 2: 179 short-read samples matched to the 154 assemblies in Sheet 1. All the short-read samples are matched to assemblies from the same participant, but not necessarily from the same timepoint. "SampleID" and "Original_ID" refer to the short-read sample. "ASM_SampleID" and "ASM_Original_ID" are the short-read and long-read samples used to generate the hybrid assembly in "ASM." One short-read sample from the same participant and same timepoint was sequenced twice: MFS-635 and MFS-878.

Supplementary Data 3: **Baseline and longitudinal samples used for analyses.** Sheet 1: 395 participants with baseline short-read WGS samples (within the first 2 weeks of enrollment) and F2 ≤ 0.03. Sheet 2: 310 participants, each with two short-read WGS samples. 285 of the participants have identical lineages and F2 ≤ 0.03 for both samples; these participants have a value of 1 in the "PassQC" column. 25 participants have mixed lineage samples for at least one timepoint; these participants have a value of 0 in the "PassQC" column.

Supplementary Data 4: **Training and new data for the logistic regression error model.** Sheet 1: 802 variant calls for the training data with freebayes VCF metadata, the features passed into the error model, and a column "Real" for whether or not the variant call was real and also detected from the personal genome. Sheet 2: 2,366 variant calls from the samples without personal reference genomes. Each variant call has freebayes VCF metadata, the features for the error model, and columns for the predicted probability that the variant call is real ("predicted"

medRxiv preprint doi: https://doi.org/10.64898/2026.06.16.26355818; this version posted June 19, 2026. The copyright holder for this preprint (which was not certified by peer review) is the author/funder, who has granted medRxiv a license to display the preprint in perpetuity.
It is made available under a CC-BY 4.0 International license .

column) and a predicted binary classification of real or not real using the selected threshold of 0.67 (“pred_class” column).

Supplementary Data 5: Unfixed and Fixed Variant Calls for all samples. Sheet 1: Unfixed variant calls for 285 longitudinal WGS samples. Sheet 2: Unfixed and fixed variant calls for 285 longitudinal WGS samples. Sheet 3: Unfixed variant calls for 395 baseline WGS samples. Sheet 4: Unfixed and fixed variant calls for 395 baseline WGS samples. Sheet 5: Unfixed variant calls for all 738 WGS samples. Sheet 6: Unfixed and fixed variant calls for all 738 WGS samples.

Supplementary Data 6: Enrichment results of unfixed substitutions. Unfixed variant enrichment results across regions (genes or intergenic regions) for all baseline (Sheet 1) and follow-up (Sheet 2) WGS samples. For both sheets, p-values for synonymous and non-synonymous mutations are given separately, along with p-values for the combined set. Both original and Bonferroni-corrected p-values are given.

Supplementary Data 7: Unfixed indels. All 278 unfixed indels identified across 738 WGS samples, including phase variant annotations. A phase variant is defined as an insertion or deletion of one unique nucleotide from a repeated sequence of the same nucleotide at least 7 times.
